# Collective-Goal Ascription Increases Cooperation in Humans

**DOI:** 10.1371/journal.pone.0064776

**Published:** 2013-05-21

**Authors:** Panagiotis Mitkidis, Jesper Sørensen, Kristoffer L. Nielbo, Marc Andersen, Pierre Lienard

**Affiliations:** 1 Department of Culture and Society, Aarhus University, Aarhus, Denmark; 2 Interacting Minds Center, Aarhus University, Aarhus, Denmark; 3 Center for Advanced Hindsight, Duke University, Durham, North Carolina, United States of America; 4 Department of Anthropology, University of Nevada, Las Vegas, Nevada, United States of America; 5 MindLab, Aarhus University, Aarhus, Denmark; 6 Centre for Human Evolution, Cognition and Culture, British Columbia University, Vancouver, Canada; Hungarian Academy of Sciences, Hungary

## Abstract

**Background:**

Cooperation is necessary in many types of human joint activity and relations. Evidence suggests that cooperation has direct and indirect benefits for the cooperators. Given how beneficial cooperation is overall, it seems relevant to investigate the various ways of enhancing individuals' willingness to invest in cooperative endeavors. We studied whether ascription of a transparent collective goal in a joint action promotes cooperation in a group.

**Methods:**

A total of 48 participants were assigned in teams of 4 individuals to either a “transparent goal-ascription” or an “opaque goal-ascription” condition. After the manipulation, the participants played an anonymous public goods game with another member of their team. We measured the willingness of participants to cooperate and their expectations about the other player's contribution.

**Results:**

Between subjects analyses showed that transparent goal ascription impacts participants' likelihood to cooperate with each other in the future, thereby greatly increasing the benefits from social interactions. Further analysis showed that this could be explained with a change in expectations about the partner's behavior and by an emotional alignment of the participants.

**Conclusion:**

The study found that a transparent goal ascription is associated with an increase of cooperation. We propose several high-level mechanisms that could explain the observed effect: general affect modulation, trust, expectation and perception of collective efficacy.

## Introduction

Cooperation is an interaction that benefits mutually all agents involved. In cooperation the aggregate benefit to participants is greater than their aggregate contribution. Cooperators exhibit behavior that implies the acceptance of personal costs in order to engage in a joint activity that they expect will bring benefits exceeding these costs [1], [2], [3].

We know that there are different categories of variables that can enhance or decrease cooperative behavior (both in the lab and in the field): (1) *contextual variables* including payoffs, number of prior interactions, amount of common knowledge shared between individuals, diversity of agents in interaction, modality of production, inherent scarcity of the collective good produced, size of the group, size of the total collective benefit, and presence or not of rules organizing the collective action and allocation of the benefit of the cooperation, (2) *psychological or systemic variables* such as beliefs, economic training, experience, degree of friendship and relatedness, altruistic propensities, (perception of) effort applied to the task, expectation about how predictable is the flow of resource, degree to which the agents share an understanding of the collective action, size of the temptation to free ride, and individuals' risk aversion, and (3) *design variables* comprising how communication is structured, degree of consensus, intensity of moral suasion, and rebates [4], [5].

Our social life affords many dilemmas typically exhibiting conflicts between short-term individual and long-term collective interests. Typical social dilemmas have been formalized as the *Public Goods Dilemma*, the *Resource Dilemma*, and the *Commons Dilemma* [6], [7], [8]. Depending on the specifics of the social dilemma, various solutions exist to offset the potential of negative outcome: creation of third-party institutions or legal and regulatory frameworks, full or partial privatization of the resource, institution of responsibility principles and coordinated punishment mechanisms [5], [8]. In a situation of social dilemma, how can voluntary cooperation [9], [10] be enhanced when relying on punishment and sanctioning mechanisms or other costly third-party apparatus is not an option?

Among the psychological and contextual factors influencing cooperation, the level of trust social agents feel toward others is a determinant factor [11], [12], [13]. Social agents might clearly understand that cooperation would be beneficial in the long run. They might be willing to take a certain amount of risk by delaying an immediate benefit for a greater future prospect given what they make of the likelihood of success in a set of prevalent conditions. But if they do not trust that the outcome of the cooperation will be mutually beneficial, they probably will not take the risk to cooperate [14].

Our experiment tests if a specific modification of the modality of interaction – *goal-* v. *process-ascription* – has an impact on levels of trust and hence, on the participants' willingness to cooperate with each other later in ulterior interactions. Teams of participants were first being asked to follow a set of specific instructions organizing their participation in a collective sequence of actions. In one condition, the *transparent*, participants were being shown the goal/result of their collective action (process), while, in the other condition, the *opaque*, participants were kept unaware of the goal/result of their interactions. Thereafter, they played a public goods game. Our prediction was twofold: (i) participants in the transparent condition will be more cooperative than the ones in the opaque condition and (ii) that difference in cooperative levels will correlate with participants' expectations about other group members' willingness to cooperate. We also looked for effects on participants' emotion.

## Materials and Methods

### Ethical Statement

This experiment was approved by the De Videnskabsetiske Komiteer for Region Midtjylland. Skottenborg 26, 8800 Viborg, Denmark, DK. All participants provided written informed consent.

The participants have given written informed consent, as outlined in the PLOS consent form, to publication of their photograph.

### Participants

A total of 48 (24 females, 24 males; age: 19–30) students from Aarhus University in Denmark participated in the study, in 12 groups of 4 participants each. Exclusion criteria for participation were: showing up late to the laboratory or arriving too early and hence waiting a long period of time, and arithmetical deficiency. We controlled that the 4 participants comprising a group had never met prior to the experiment.

### Materials

#### Economic experiment and questionnaire

We analyzed the effect of the transparent goal ascription in a collective action on individuals' decisions in a standard linear one-shot public goods game with real monetary stakes. Linear public good experiments are used for examining the willingness of individuals to overcome collective action problems [7]. In the game, two subjects, interacting anonymously, play the role of investors in a common project after having been endowed with a fixed set of assets (Table 1). Both participants make their decision simultaneously, anonymously, and without previous negotiation. They have the option of investing -or not- in a common project in which the money invested is multiplied by a factor of 1.5. The experimenter collects the contributions, sums them up, multiplies the amount, and allocates it equally among the subjects.

**Table 1:** The public goods game.

	C	D
C	150/150	75/175
D	175/75	100/100

The payoff (in DKK) of each person is given as: 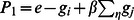 where: 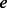 = initial endowment in “tokens” not varying across subjects, 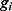 = tokens subject i contributes to the group public good account, 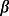 = marginal payoff to each individual from the public good, and 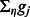 = the sum of the n individual contributions to the public good.

Thus, if both subjects invest the whole amount they get the maximum as a group. The unique Nash Equilibrium of the game is zero contribution from both subjects, since a player could potentially gain more by defecting if the game partner were to contribute. The game reflects the tension between individual incentives and collective benefits. Both investors are caught in a dilemma; if one player invests, and the other defects, she may end up with less than she had at the start of the game. If both cooperate and invest their money, both benefit equally from the cooperation. Substantial evidence shows that culture has an influence on cooperation, mainly in the presence of reward or punishment opportunities [15], [16]. Moreover, we know that individuals choose cooperation strategy more frequently when they have the feeling of participating in a groups' endeavor. [17].

### Design

**Figure 1:**
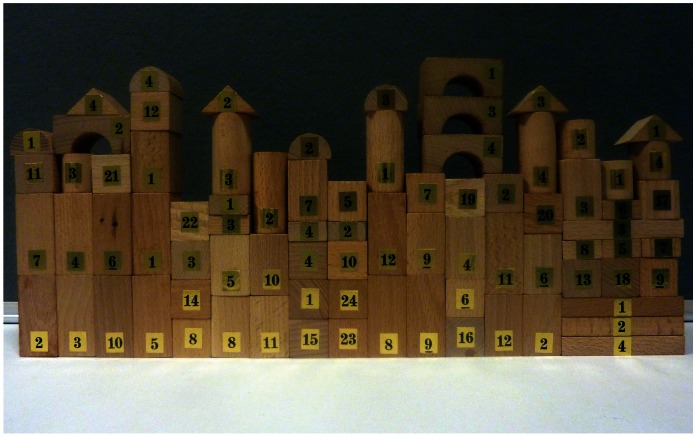
Collective goal.

Participants were randomly assigned to either one of two conditions: *transparent* or *opaque* (6 groups of 4 participants were assigned to the transparent condition, 3 male- and 3 female-only groups, and 6 groups of 4 participants in the opaque condition, 3 male- and 3 female-only groups). In the transparent condition the participants were asked to follow a set of instructions displayed sequentially on a computer screen instructing them when and how to participate. They were also shown what the final outcome of their interaction would be: a building made of wooden blocks of different shapes (Fig. 1). In the opaque condition, participants were asked to follow the same set of instructions as in the *transparent* condition but were not made aware of the end-result of the actions of the group. After the manipulation, the participants played a two-players public goods game. They were told that they would be playing with one of their previous teammates for real monetary stakes, and that they had to decide if they wanted to invest money (and how much) in the common project. Participants were then asked to provide their estimation of the other player's contribution and after completion of the game, they were administered a questionnaire. Participants' basic demographic information was collected. Other specific questions addressed participants' level of group participation in their everyday lives, and their impressions of the manipulation task. All instructions and questions were provided in Danish (the native language of the participants), and a Danish assistant, blind to the hypotheses, conducted the experiment. We used 4 participants in the manipulation process in order to assure that anonymity would not be perfectly revealed in the following economic game and so we controlled for attraction effects.

### Procedure

Participants were recruited via flyers among the student population of the university. Participants were invited to take part in a behavioral economic experiment. A few days before the experiment was to take place, the experimenter contacted each potential participant by phone to invite him/her at a specific time to the laboratory facilities. After the phone contact, the participants received an email reminding them of the time and location where the experiment was to take place. They were also reminded of the nature of the experiment and asked to send a signed written informed consent via email before participation.

**Figure 2:**
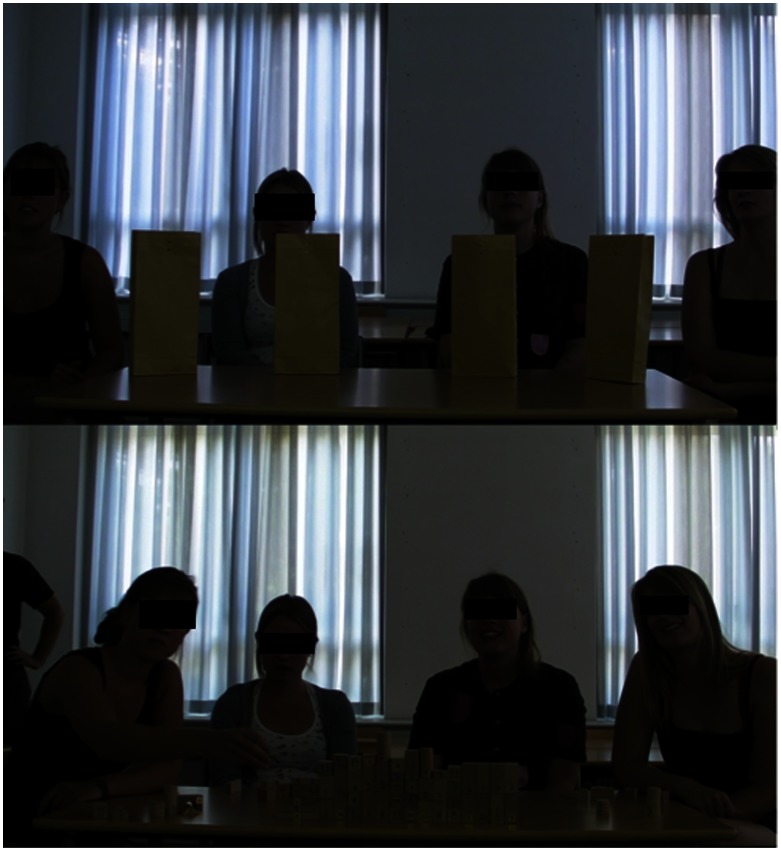
Participants during the manipulation task.

Upon arrival to the laboratory the participants were assigned to distinct waiting rooms in order to avoid any communication prior to the experiment. They were then led to another room, where they were asked to sit next to each other, facing a table and a large monitor on which the instructions to follow would be displayed. In front of each participant was a paper-bag (Fig. 2) containing 20 building blocks of different shapes (80 blocks, each to be manipulated once by one of the teammates, for a total of 20 actions per participant). The participants were told that their objective was to build together a construction, using the wooden blocks, following the instructions sequentially displayed on the monitor (Fig. 1,2).

The participants where not told whether or not to communicate with each other. Which participant's turn it was and which block was to be placed in a particular position were systematically specified in the instructions displayed progressively on the monitor. The participants were also instructed not to touch any of the other players' blocks. The procedures were identical in both conditions, but for the fact that the participants in the transparent condition could see displayed on the monitor the final product of their actions, whereas in the opaque condition the participants could not. The sequence of instructions to be systematically followed controlled for the risk of social loafing effects.

At the end of the phase of manipulation, each one of the four participants was immediately isolated in individual rooms. On a desk in each of the four rooms were a stack of 5 sheets of paper and a pen. The first sheet presented the instructions for the public goods game, followed by a control test that required from them to solve simple examples of the economic game. The third sheet was the decision form, on which they were instructed to write the amount they wanted to invest. In the fourth sheet, participants were asked to write their estimation of the amount that the other player would invest in the common project. Finally, the last sheet was the questionnaire (41 questions in total). When done with the questionnaire, the participants were asked, one by one, to come to the control room, where they were debriefed in isolation (hence preserving the anonymity of all participants), received their respective payment, and asked if they wanted to get more information or to provide any feedback. The experiment lasted about 45 minutes: 11 minutes for the building interaction phase and 30–34 minutes for the economic game and questionnaire. The minimum amount paid was 75 DKK (≈10 euros), and the maximum was 175 DKK (≈23.5 euros).

## Results

### Cooperation

**Figure 3:**
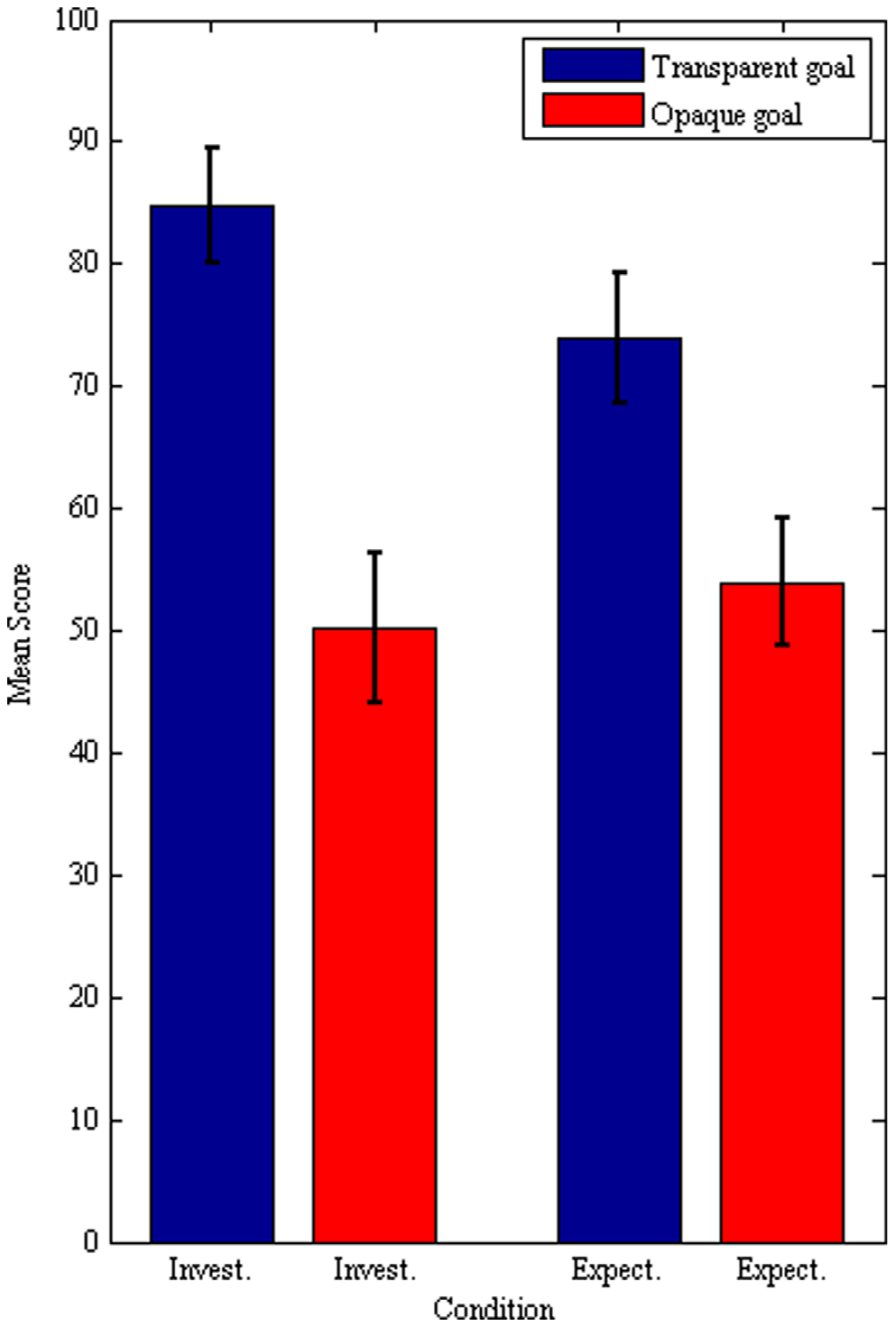
Investments and expectations in the public goods game. Each observation represents the amount invested or expected to be invested. Participants in the transparent condition show significantly higher investment levels, and subsequently higher expectations about the investment of the other player (n = 48).

Our main prediction was that the participants in the transparent condition would show higher levels of cooperation than the participants in the opaque condition. In fact, our data show that the transparent collective-goal ascription increases cooperation considerably (Fig. 3, Table 2). A one-way between subjects ANOVA was conducted to compare the effect of action type on investment in the two conditions. There is a significant effect of action type on investment at the *p*<.05 level: *F*(1, 46) = 9.88, *p* = .003. Because the data did not fully satisfy the assumption of normality, a one-way Kruskal-Wallis test was conducted. The test confirms the results by showing a significant difference in investment as a function of action type (Fig. 3). The difference between the rank totals of 30.1 (transparent) and 18.9 (opaque) is highly significant, H(1) = 9.606, p = .0019, ω2 = .184. The effect size associates with this difference, as measured by η^2^, was .18, which is a large effect according to Cohen's criteria [18]. These differences in the distribution of cooperation are reflected in higher average and median cooperation levels for participants in the transparent condition (Table 2).

**Table 2:** In the transparent condition participants' investment is 34.6% higher than in the opaque condition, and the median investment is 100, compared to a median of only 50.

	Investment		Expectations	
	Transparent	Opaque	Transparent	Opaque
Mean average investment	84.8	50.2	74	54
Median average investment	100	50	100	50
Standard deviation of transfers	35.9	38.6	43.3	36.8
Number of observations	48	48	48	48

In the transparent condition participants' expectations are 20% higher than the opaque condition, and the median on expectations is 100, compared to a median of only 50.

### Expectations

Our second prediction about participants' expectations was also confirmed (Fig. 3, Table 2). The investors' expectations about what the other player will do (reciprocate or not) are following the investment/cooperation levels (Fig. 3). To compare the effect of action type on estimation in the transparent and opaque conditions a one-way Kruskal-Wallis was performed. The non-parametric test shows a significant effect of action type on estimation at the *p*<.05 level: χ^2^(1, *N* = 48) = 4.35, *p* = .037. Investment and expectation are highly correlated in the transparent condition: *r*(44) = .76, *p*<.001; and in the opaque condition: *r*(44) = .75, *p*<.001.

### Emotions

**Figure 4:**
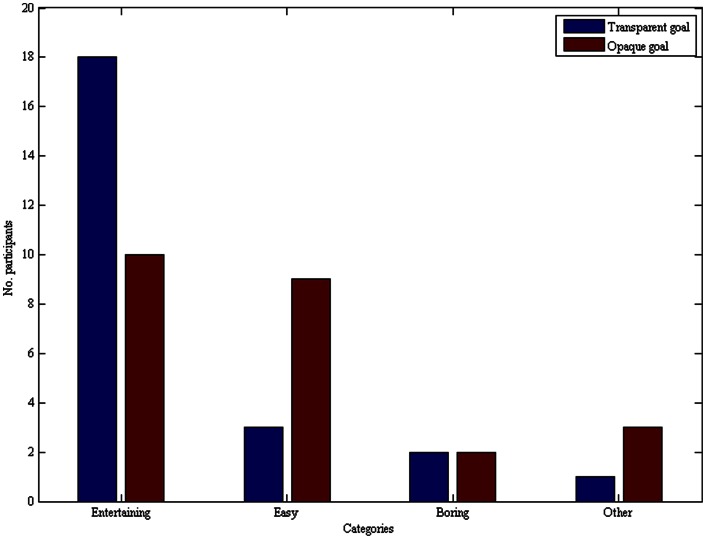
Synchronization of behavior and emotions. Participants in the transparent condition have reported happier emotions about their participation, whereas participants in the opaque condition reported mixed emotions.

The questionnaire provides some interesting data supporting the previous findings of the main effect of transparent collective-goal ascription on cooperative behavior (Fig. 4). In the transparent condition, 75% of the participants reported that the process was entertaining, which is significantly more than would be expected by chance, exact binomial *p*(two-tailed) = .022. In contrast, the opaque condition only elicited an “entertaining” response from 42% of the participants, which is not significantly different from what chance predicts, exact binomial *p*(two-tailed) = .541. Only 13% found the transparent task easy, which is significantly less that would be expected by chance, exact binomial *p*(two-tailed) = .0003; while 38% found the opaque task easy, which is not significantly different from chance level, exact binomial *p*(two-tailed) = .308. Effects of memory, gender or level of everyday-life group participation on investment, independent of action type, were not significant.

## Discussion

The research presented here belongs to a long history of inquiry into the benefits and consequences of the rationalization of administrative processes in modern states with formal institutions. Process-ascription warrants equality of access and treatment in complex modern institutions. But such ascription seems to have also some unintended consequences. In the first half of the 20^th^ century, social scientists such as Durkheim and Weber had already addressed the inherent advantages and pitfalls of stipulating administrative and bureaucratic processes [19], [20], [21]. A typical tendency associated to process-ascription is the disconnection between rigidly prescribed processes and their ultimate intended goals.

In the experiment, in one condition we recreate such a disconnection between process and goal while in the other condition we reinforce the perception that individuals interact in a logical and stepwise manner toward a stipulated goal. The experimental data collected seem to support the idea that, all else being equal, a minimal manipulation, *i.e.* the clear and sustained ascription of a goal, can have a tremendous impact on the reinforcement of lasting cooperative units. Below, we address the question of how coordinated action, with a clearly identifiable goal, leads to higher levels of cooperation. We also discuss potential psychological mechanisms explaining our results.

In the transparent condition, participants have the experience of a shared collective goal, unlike in the opaque condition. It has been shown that having a readily accessible goal in mind enables agents to address at the same time other competing goals, such as reputation monitoring [22]. When a goal is publically stipulated, social agents are more willing to coordinate their actions because they care to be perceived as good prospects for further interactions. However, we may still wonder why in our specific manipulation the ascription of a goal has a long-lasting impact on the participants. The participants follow a strict sequence of actions allowing no individual initiative. Yet, later in the economic game their willingness to cooperate increases. So, how does interaction give rise to the perception that coordination and cooperation are occurring eventually leading to more willingness to cooperate in ulterior interaction? One potential answer to that puzzle might be that the collective goal ascription creates the illusion of a constructive interaction, allowing for the reframing of the individuals' prescribed actions as part of a cooperative scheme. In both conditions, the participants evaluate the situation, but their respective evaluations differ in how individual contributions are perceived. In the transparent condition, the contribution of the participants is evaluated not just as response to a set of instructions, as in the opaque condition, but as actual contribution to the fulfillment of a collective goal. This leads participants to reconstruct what happened, in terms of a collective goal, re-conceptualizing it as an actual case of cooperation. By contrast, in the opaque condition individual actions seem harder to be integrated into a collective goal-structure hence are plausibly less likely to be interpreted as cooperative.

Studies indicate that the coordination among work group members is enhanced if the members have a shared mental model of the procedures to be followed [9], [10], [23], [24], [25]. Coordinated behavior occurs when all agents perform actions that correspond to the necessity of satisfying a goal conjointly. The visually transparent goal facilitates the perception of coordination. Subsequently, coordination impacts how cooperative the participants will be, as they probably regard the other participants as coordinators having a shared goal and similar utility about the known outcome. In a theoretical vein, we speculate that transparent goal ascription also contributes to a lower prediction error rate [26], [27]. Transparent goal leads to enhanced cooperation because the available information is more advanced about the choice options available, as opposed to guesses when the outcome is unclear.

The amount that participants contribute to the public goods game is our basic measure of cooperation levels. The game takes place after the phase of manipulation indicating a carry-over of the effect to the new and different task. We propose two main alternative explanations for our findings. (1) The experimental manipulation induces particular types of affects moderating participants' overall dispositions to cooperate. Contrary to what would occur when no clear objective is identified, the ascription of a goal might induce positive affects in participants by the sheer fact that it facilitates the integration of the entire experimental activity. The positive affect borne out of the relatively more pleasing experience would eventually influence how increasingly other-regarding participants become. Conversely, the absence of a goal ascription could make the overall experience less pleasing in the opaque condition eventually moderating the level of willingness to interact positively with other social agents. (2) We identify another likely mechanism that could be at play here: the level of trust that the participants feel toward one another. The participants in the transparent condition seem to have identified their teammates as good cooperators and to be confident and willing to invest in further cooperation despite the inherent risk of being exploited. Therefore, we argue for a model of trust as enhancing cooperative behavior lasting in time [28], [29].

Social psychologists have identified this link between trust and cooperation in the “goal/expectation theory” [30]. Mutual trust is essential to good cooperation and cooperative behavior arises when both parties have a goal specifying their mutual cooperation as well as a common expectation that the other will also cooperate. Ascription of a transparent goal in a group of people creates not only shared goals but also aligns the expectations concerning the group's performance. In both conditions, we see that participants have coordinated their expectations with the others of the group, when it comes to cooperative investment. High investments are followed by high expectations and *vice versa*. Given how our experiment was designed, we cannot decide if the investment decisions are based on expectations or if the participants rationalize their evaluations of others' investment based on what they have invested in the first place [31].

Finally, we believe that a transparent collective-goal creates perceptions of collective efficacy. Participants in the transparent condition have the impression of a collective success. In contrast, in the opaque condition, participants do not have a perceptible anchor to relate to and therefore they seem to have difficulty forming a collective unit dedicated to achieving a goal [32]. This interpretation is supported by the correlation between participants' investments in the game with the data about their feelings during the “building” process, as reported in the questionnaire. Participants seem to align or synchronize their emotional states (Fig. 4). In social interaction, emotions can pass from one individual to another by imitation, mimicry, and empathy. Emotions are indicators of a re-framing process. We see that the more cooperative participants are the ones who found the process more entertaining, whereas the less cooperative participants report more mixed emotions. The transparent condition might lead to individual reconceptualization of having reached collectively a goal.

Cooperation characterizes many human social interactions. We may wonder, as Axelrod and Hamilton did, “under what conditions will cooperation emerge in a world of egoists without central authority?” [33] Our results speak to the great importance of the ascription of transparent goal in creating and enhancing cooperation. We have not fully provided a cognitive model that could explain the observed effect but we were able to identify new areas of research that have the potential to refine our understanding of cooperation. The study suggests that ascription of a transparent collective goal functions as a top management mechanism that can significantly enhance cooperation and facilitate overcoming the problem of freeriding in a public-goods dilemma. As we have shown, a refined knowledge of the goal of a collective endeavor has an impact on the agents' disposition to engage in cooperation. Ultimately the capacity to foster thoughts about collective goals might have played an important role in the build-up of wider cooperative networks not explicable by kin selection or direct reciprocity.
